# Colon Sparing Endoscopic Full-Thickness Resection for Advanced Colorectal Lesions: Is It Time for Global Adoption?

**DOI:** 10.3389/fonc.2022.967100

**Published:** 2022-07-13

**Authors:** Zhong-Wei Wu, Chao-Hui Ding, Yao-Dong Song, Zong-Chao Cui, Xiu-Qian Bi, Bo Cheng

**Affiliations:** ^1^ Department of Gastrointestinal Surgery, The First Affiliated Hospital of Zhengzhou University, Zhengzhou, China; ^2^ Department of Emergency Surgery, The First Affiliated Hospital of Zhengzhou University, Zhengzhou, China

**Keywords:** colon cancer, endoscopic therapy, endoscopic submucosal dissection, endoscopic full-thickness resection, gastrointestinal cancer

## Abstract

The majority of colon lesions are <10 mm in size and are easily resected by endoscopists with appropriate basic training. Lesions ≥10 mm in size are difficult to remove technically and are associated with higher rates of incomplete resection. Currently, the main endoscopic approaches include endoscopic mucosal resection (EMR) for lesions without submucosal invasion, and endoscopic submucosal dissection (ESD) for relatively larger lesions involving the superficial submucosal layer. Both of these approaches have limitations, EMR cannot reliably ensure complete resection for larger tumors and recurrence is a key limitation. ESD reliably provides complete resection and an accurate pathological diagnosis but is associated with risk such as perforation or bleeding. In addition, both EMR and ESD may be ineffective in treating subepithelial lesions that extend beyond the submucosa. Endoscopic full-thickness resection (EFTR) is an emerging innovative endoscopic therapy which was developed to overcome the limitations of EMR and ESD. Advantages include enabling a transmural resection, complete resection of complex colorectal lesions involving the mucosa to the muscularis propria. Recent studies comparing EFTR with current resection techniques and radical surgery for relatively complicated and larger lesion have provided promising results. If the current trajectory of research and development is maintained, EFTR will likely to become a strong contender as an alternative standard of care for advanced colonic lesions. In the current study we aimed to address this need, and highlighted the areas of future research, while stressing the need for multinational collaboration provide the steppingstone(s) needed to bring EFTR to the mainstream.

## Introduction

The flexible endoscope was originally designed as a diagnostic tool for examining the lumen of the gastrointestinal (GI) tract, now known as the ‘first space’ (1). The utility of expanding the operative field to this ‘second space’ has been highlighted by advances in interventional endoscopic techniques such as the development of a working channel, access to the peritoneal cavity and more recently, access to extra-luminal organs such as the gallbladder and thyroid gland (2–4). Access to the submucosal layer – also known as the ‘third space’ – and to the subserosal layer – known as the ‘fourth space’ –has highlighted the potential of interventional endoscopy in reducing the need for invasive surgical interventions for the management of many luminal lesions (5).

Endoscopic mucosal resection (EMR) and endoscopic submucosal dissection (ESD) are two well established endoscopic resection techniques for lesions involving the mucosal and superficial submucosal layers (6, 7). However, EMR and ESD can be challenging as there is still a considerable rate of adverse events, particularly perforation and bleeding. Furthermore, larger lesions cannot reliably be removed resulting in incomplete resections, and dense fibrosis results in non-lifting, difficult-to-treat lesions (8, 9). ESD is technically challenging and burdened by longer procedure times and higher costs. It should therefore be restricted to lesions suspicious for high-grade dysplasia or early invasive cancer.

Endoscopic full-thickness resection (EFTR) is a latest addition to endoscopic resection techniques which is developed to overcome the limitations of EMR and ESD (10, 11). It involves the complete resection of the mucosa to the muscularis propria and is very useful for the treatment of difficult-to-resect lesions, e.g., recurrence with scar formation after previous endoscopic resections or even for associated lymph nodes resections (12). There are two approaches to EFTR i.e., exposed and non-exposed EFTR. In exposed EFTR, first the full-thickness resection is performed, with subsequent closure of the mucosal defect (mainly used for upper GI luminal subepithelial lesion). In non-exposed EFTR, serosa-to-serosa apposition is achieved, and then full thickness resection is completed with the assistance of a cap-mounted clip. However, it is challenging to safely and efficiently close the large defects resulting from EFTR. Successful closure of large defects is critical for the use of the EFTR procedures. Endoclips were used as the earliest treatment method for the endoscopic closure of GI perforations and are still a common tool for defect closure. Different types of endoclips with reliable rotation are available widely and are usually effective for the closure of small defects (<1 cm) but are less effective for the closure of larger defects (>1 cm).

Different suturing devices/systems have been developed for EFTR such as over-the-scope clips (OTSC) (Ovesco Endoscopy AG, Tubingen, Germany) and Overstitch (Apollo Endosurgery, Austin, TX, USA). OTSC is a clip-type full-thickness suturing device but is still unable to close defects larger than 2 cm. Several studies have reported the usefulness of the device for closure of fistulas, iatrogenic perforations and anastomotic leakage after surgery (13–16). The overstitch is not available worldwide and even in China. More recently Liu et al. designed the new technique of “Kissing Suture” for closing large GI wall defects that remained after EFTR. The successful use of the “Kissing Suture” method for endoscopic gastroplasty and NOTES gastroenterostomy (anastomose the jejunum and stomach) has been described in a human setting and has proven to be feasible and efficient (17–19). Since the introduction of EFTR with full-thickness resection device (FTRD) into clinical practices, there has been an increasing number of published studies on EFTR using the FTRD system in the colorectum (20, 21).

If the concerns of infection and intraperitoneal diffusion of tumor cells are solved, advanced complicated lesions such as gastrointestinal stromal tumors (GISTs) would be a good candidate for EFTR. Flexible endoscopy has opened up new frontiers for surgeons and endoscopists. As the armamentarium of interventional endoscopy expands and the ability of endoscopists to perform advanced interventions safely fosters an inevitable step forward that will involve the integration of new technology with innovative and creative thinking.

## Endoscopic Full-Thickness Resection

EMR for adenomas or early colorectal carcinomas with severe fibrosis have an increased risk of perforation and other adverse events. The perforation rate of ESD for lesions with severe submucosal fibrosis also remains high. Endoscopic full-thickness removal of luminal GI lesions is an efficient non-surgical development for en bloc resection for advanced adenomas (22, 23). Endoscopic resection of luminal GI neoplasms has traditionally been limited to the mucosa and submucosa due to the lack of adequate closure techniques. The introduction of the OTSC and the research on natural orifice transluminal endoscopic surgery (NOTES) paved the way for transmural endoscopic interventions (14, 24). In 2014, two groups separately reported the first clinical cases of EFTR using a OTSC device (25, 26). Since the approval of the FTRD (in 2014), multiple studies on colorectal EFTR have been published (27). Lesions <20 mm in diameter may be amenable to resection with an adapted OTSC cap, in which the lesion is pulled into the cap with retraction forceps resulting in invagination of the colon wall followed by OTSC deployment.

Zwager et al. reported the results of the Dutch colorectal EFTR registry where the data was prospectively collected from 20 hospitals and covered a total of 367 EFTR procedures (28). The procedure technical success was 83.9% and the R0 resection rate was 82.4%. Adverse events were noted in 9.3% of cases, with 2.7% of patients required emergency surgery. Recurrent or residual lesions rate was 6.4%. Interestingly, the Dutch colorectal EFTR data included a sizeable subgroup of patients (n = 221) undergoing EFTR for T1 carcinoma. A recent retrospective study described a high diagnostic value of EFTR for T1 carcinomas, allowing patients to be assigned to the best treatment strategy (29). The Dutch registry comprised 150 patients who had “secondary” EFTR after malignant polyps were not completely resected. According to Kuellmer et al., the vast majority of these patients were classified as “low risk” after EFTR, and surgery was therefore not deemed necessary (30). According to both studies, EFTR of post-polypectomy scars appears to be a very good alternative in these cases.

Guo et al. presented 23 cases of exposed EFTR followed by defect closure using the OTSC method. The procedure technical success rate was 100% and delayed perforation was not observed at the 3-month mark (31). Tumor size ranged from 6–20 mm. Histopathological findings showed that one patient (4%) was high-risk GIST, 18 (78%) were very low-risk GIST, and 4 (17%) were leiomyomas. The main reported complication was localized peritonitis which occurred in two patients (9%). Zhou et al. and colleagues reported 26 EFTR cases for gastric GIST originating from the muscularis propria. R0 resection was achieved successfully in all tumors with a median size of 28 (range 12–45) mm. Full-thickness resected stomas was successfully closed using endoclips in all cases including the one where the defect was larger than 30 mm (32).

**Figure 1:**
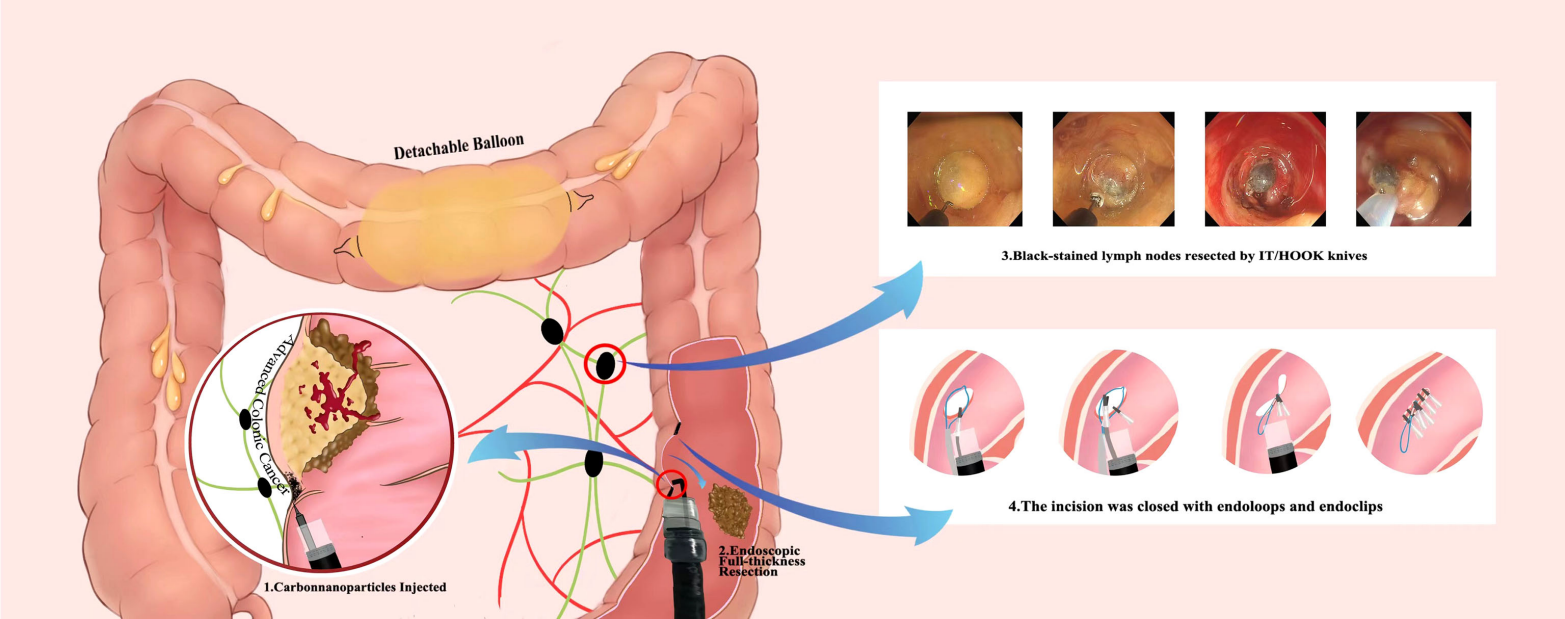
Infographics of endoscopic full-thickness resection and lymphadenectomy for advanced colonic cancer as described by Liu et al.

Recently, Liu et al. reported EFTR of colonic adenocarcinoma and lymphadenectomy in a 60-year-old man (33). Due to multiple prior abdominal surgeries, he was not considered a candidate for another intra-abdominal operation and therefore, endoscopic resection was recommended for him. A detachable balloon was inflated and placed in the transverse colon before the procedure to prevent contamination of the intraoperative field. EFTR of the colonic lesion was then completed successfully. An endoscope was introduced *via* the mucosal defect, to observer the lymph nodes. The lymph nodes were observed at 2-3 cm from the defect area and removed endoscopically. The large full-thickness defect was then closed using endoloops and endoclips **(** **Figure 1** **).** No adverse event was observed in the patient at the 12-month follow-up visit.

This case demonstrates the EFTR of a colorectal cancer and a lymphadenectomy. Although this case was striking, and the patient appears to be disease-free. The authors may simply gotten lucky in that the nodes were all easy to locate, they were adjacent to the original lesion, and could be resected easily. The majority of colorectal cancer surgeries involve an extended search for adenopathy and removal of all perilesional lymph nodes. It is easy to visualize how other benign or malignant lymph nodes could have gone unnoticed; if that were the case, there would have been far less to celebrate.

## Endoscopic Full-Thickness Resection – Indications, Closure Techniques, Complications and Limitations

In clinical practice, non-lifting lesions with extensive submucosal fibrosis and lesions involving difficult anatomical locations (appendiceal orifice or diverticula, and subepithelial tumors) are the main indications for EFTR. Moreover, EFTR is gaining attention as a credible diagnostic tool and alternative therapeutic option for T1 colorectal cancer, because it can provide high quality pathological specimens and precise histological risk assessment. GIST has been found to be a good indication for EFTR as well.

**Figure 2:**
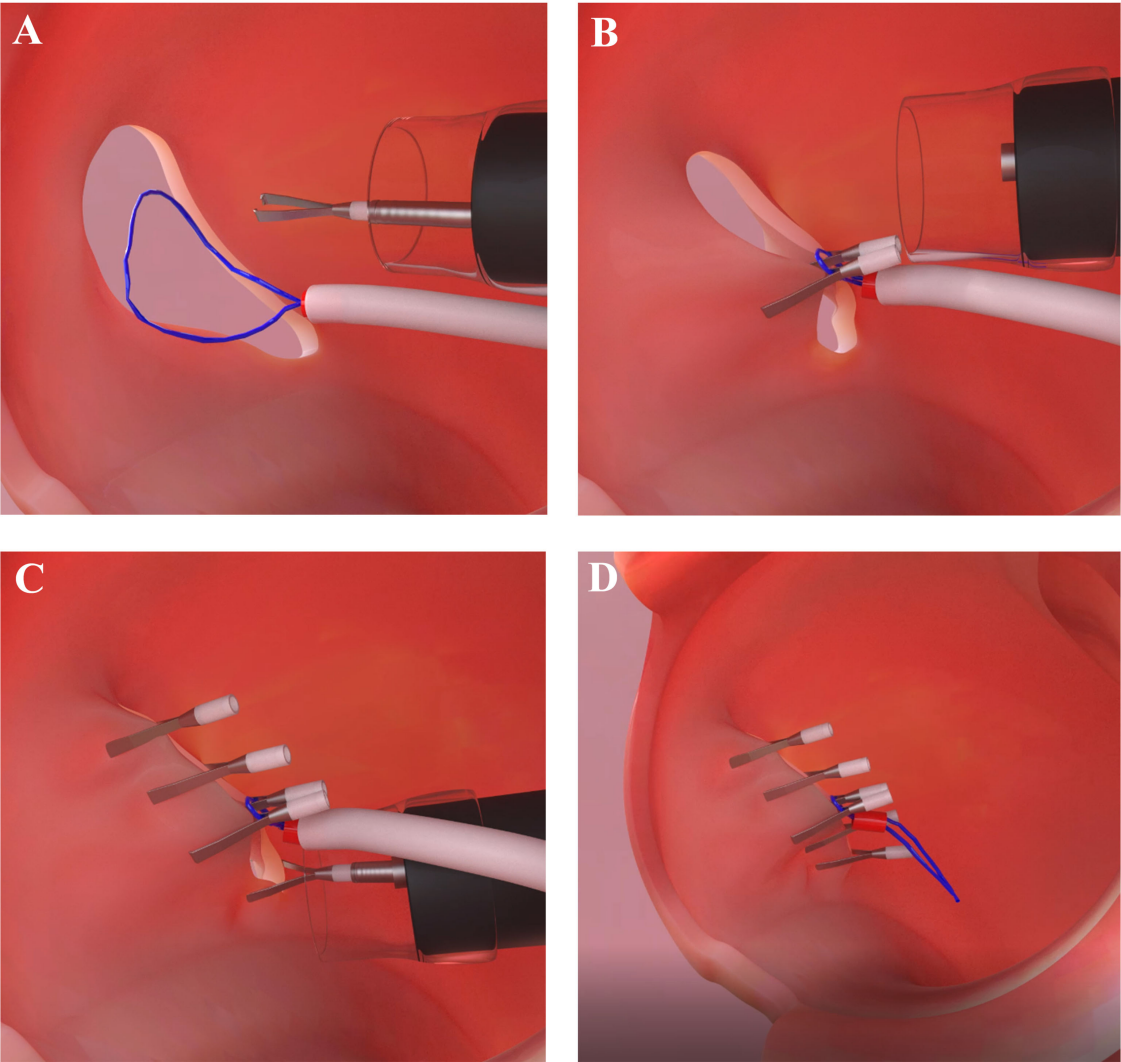
Illustration of the ‘‘Kissing Suture’’ method. **(A)** Nylon loops along with endoclip is used to anchor the two edges of the defected wall. **(B)** Nylon loops is fixed along with endoclip. **(C)** Additional clips are used to complete the linear incision closure. **(D)** Overview of the defect closure using nylon loops and endoclips.

To date, several full-thickness endoscopic closure methods have been developed using endoclips with or without endoloops, as well several suturing devices, including the OTSC system or Overstitch is the most commonly using tool in clinical settings (34–38). The first published report of the use of endoclips to close perforations after resection of a gastric leiomyoma was in 1993 (39). Subsequently, multiple studies have demonstrated the successful use of endoclips for the closure of iatrogenic perforations resulting from EMR and ESD (40). Endoscopic clipping is now accepted as the most effective, popular, and widely available method for the closure of GI wall perforations (40, 41). Following the development and success of the use of endoclips, an endoscopic suturing methods for EFTR has been used to avoid the need for surgical intervention. However, endoclips can grasp only the mucosal layer, and closure of large defects is challenging if the defect size is larger than 1 cm, thus, there is a risk of leakage when using endoclip alone closure (42). To date, numerous studies have reported the successful use of the OTSC system and Overstitch for EFTR defect closure (31, 43). The major limitations of OTSC included limitation of maximum tumor size (up to 2 cm, due to the restraint of tissues that can be sufficiently pulled into the cap), tumor position (esophagus and duodenum due to limited workspace), and restricted maneuverability for defect closure. Additionally, in China, where the majority of the EFTR procedure has been performed in the world Overstitch is not available (34). To overcome the limitations of endoclips for large defect closure, endoscopists have designed several methods in combination with endoloops based on use of a double-channel endoscope, such as interrupted suture, clip-loop, endoscopic purse-string suture methods, and so on (35–37). None of these methods are suitable for defects larger than 4 cm (44). The use of a double-channel endoscope has disadvantages. First, a double channel endoscope is thicker, rigid, and heavier; therefore, it is difficult to manipulate. Second, the endoclip and endoloops are inserted through two different channels of the same endoscope, both move synchronously with the endoscope; thus, making it difficult for clipping the nylon loops around the edge of the defect. Third, it is necessary to change to the double-channel endoscope for defect closure, whereas the resection was made with a single-channel endoscope. Fourth, the double-channel endoscopes are not available in most of the endoscopy centers in China and other countries. Because of the technical limitations and difficulties of using a double-channel endoscope as well as endoscopic suturing devices, Liu et al. designed the “Kissing Suture” method for closing large GI wall defects that remained after EFTR. The ‘‘Kissing Suture’’ method requires a single-channel endoscope with endoloops and endoclips. The loop is placed in the defect area and uses two endoclips to anchor it to the two edges of the full-thickness layer or muscle layer of the defected wall. Tightening the loop changed the defect from linear-shaped to resemble the infinity symbol (∞). To the purpose of more rapid wound healing the defected edges are set in edges-to-edges apposition at the edge of submucosa-submucosa and mucosa-mucosa, respectively; and then endoclips are used to close the complete linear incision **(** **Figure 2** **).** Benefits of the “Kissing Suture” method included, it is easy to control the endoscope because the endoloops free from the endoscope and does not interfere with clipping. Additional, endoloops and endoclips can be used to reinforce the closure of the defect and prevent postoperative leak and peritonitis. However, the mucosa may invert while mucosa and submucosa are sutured together which could impair healing. From previous experience, defect closure with endoclips by the mucosa-mucosa apposition has been shown to be effective for quicker wound healing and prevention of ongoing leakage of air and digestive juices (17). The use of a single-channel endoscope with endoloops and endoclips has the advantage of being simple to manipulate, does not require specialized equipment, and there is no technical complexity. The ‘‘Kissing Suture’’ method has a potential future for the closure of large GI tract wall defect following EFTR, NOTES procedure, and for different kinds of perforation.

Bleeding and perforation are main EFTR complications. When bleeding occurs during the procedure, the use of dedicated hot coagulation forceps and/or hot biopsy forceps to stop it immediately are recommended. However, endoscopic clips should be used to ligate the bleeding vessels when large vessels are present, or the bleeding cannot be stopped using coagulation. To avoid perforation, use the dynamic submucosal injection approach for sessile and flat lesions to separate the lesion from muscularis propria with an adequate submucosal cushion, and take preventive measures if a deep resection is performed. It is critical to be able to recognize deep cuts or perforations since they must be closed immediately using endoclips.

## Development and Training for Endoscopic Full-Thickness Resection

Gastroenterology fellows are trained to perform colonoscopy for diagnostic purposes in a straight forward fashion. It is essential to have a thorough understanding of EMR and ESD before attempting EFTR. EFTR expands upon these skills and transposes skills acquired from training in EMR or ESD (indications, proficiency in lesion identification, limitations, technique, and dealing with adverse events during the procedure). Although more advanced methods can be learned during ESD training, competency in the handling of adverse events such as perforation and bleeding is mandatory. Deconstruction of the steps involved in EFTR allows for task-oriented training. Adopting these basic principles, a credentialing process for competency in EFTR should be established as this will be essential for global adoption of this endoscopic modality.

Although there are a variety of artificial tissue and ex vivo models available, opportunities to practice on live animals that better imitate peristalsis, bleeding, and respiratory movement should be pursued. However, training specific to the colorectum remains challenging because live animal models such as porcine models do not provide adequate simulation of human colorectal tissue. Bovine specimens are good alternatives for the colorectum, particularly when training to use innovation such as the FTRD and other new resection tools. With the experience and improvements in endoscopic technology, the procedural difficulties observed with EFTR will be reduced as well. Additionally, patients with advanced adenomas who are poor surgical candidates may also benefit from EFTR.

## Future Direction and Conclusion

Traditionally, colectomy remains the treatment of choice for the management complex colorectal lesions. However, advanced endoscopic techniques have also been proposed in order to improve the accuracy of diagnosis, more precise risk assessment, and colon sparing resection of colorectal lesions. Advanced endoscopic resection techniques allow curative treatment of difficult colonic lesions and often avoids the need for surgery. However, determining the optimum resection technique (EMR, ESD or EFTR) for specific individual and lesions to maximize the efficacy, and safety while avoiding unnecessary surgical intervention remains a challenge. Although both EMR and ESD are effective and are currently preferred, a transmural approach for large lesions, if resources and expertise are available would be preferable.

Endoscopic full-thickness resection, a transmural approach, of luminal GI lesions is a new development that has been performed only in a limited manner and, in all fairness, can only be labeled as experimental. Nonetheless, interest and progress in this field are rapid and ongoing. Although EFTR technique is still developing and requires refinement, it is an outstanding procedure in terms of invasiveness. However, we should not be satisfied with the currently available numbers and retrospective studies. We must aim for large sample size prospective randomized controlled trials to compare the EFTR with other endoscopic methods with long-term follow-up data on recurrence rates and clinical outcomes. Development of reliable closure devices and establishment of appropriate indications will make EFTR more practical especially for advanced colonic lesions. This not only avoid patients to an unneeded, invasive intervention with its associated risks, but also reduces the financial burden on the patient, hospital system, and society. There is also a need for multinational collaboration and a consensus on training and credentialing pathway for EFTR, and on areas of future research necessary for widespread adoption of EFTR.

## Data Availability Statement

Publicly available datasets were analyzed in this study. This data can be found here: This is a perspective thought provoking article (not an original article). No original data was produced for this article. However, current data (non-original) collected for writing this article is available with corresponding author. It can be provided on request. (BC, E-mail: edgar2017lw@126.com).

## Ethics Statement

The study protocol was reviewed and approved by the independent ethics committee of at the First Affiliated Hospital Zhengzhou University.

## Author Contributions

Study concept and design: Z-WW and BC. Manuscript writing: Z-WW, C-HD, Y-DS, and Z-CC. Acquisition of data: X-QB, C-HD, Y-DS, and Z-CC. Schematics drawn: Z-WW. Critical revision of manuscript: BC. All authors contributed to the article and approved the submitted version.

## Conflict of Interest

The authors declare that the research was conducted in the absence of any commercial or financial relationships that could be construed as a potential conflict of interest.

## Publisher’s Note

All claims expressed in this article are solely those of the authors and do not necessarily represent those of their affiliated organizations, or those of the publisher, the editors and the reviewers. Any product that may be evaluated in this article, or claim that may be made by its manufacturer, is not guaranteed or endorsed by the publisher.
